# Integrin alpha L: structure, cellular functions, and emerging role in human diseases

**DOI:** 10.3389/fimmu.2025.1707291

**Published:** 2026-01-12

**Authors:** Xianglin Wang, Guoya Yang, Yanpei Chen, Fang Zhu, Fuming Lian, Wenzhi Shen, Dehong Luo

**Affiliations:** 1 The Third Affiliated Hospital of Zunyi Medical University (The First People’s Hospital of Zunyi), Zunyi, China; 2 Shandong Provincial Precision Medicine Laboratory for Chronic Non-communicable Diseases, Institute of Precision Medicine, Jining Medical University, Jining, China

**Keywords:** Integrin α L, intercellular adhesion molecule, biomarker, immune regulation, cancer, human disease

## Abstract

As an integral component of Lymphocyte Function-associated Antigen 1 (LFA-1), Integrin α L is crucial for the processes of leukocyte adhesion and migration. It engages in specific interactions with Inter-Cellular Adhesion Molecules (ICAMs), thereby playing a significant role in intercellular adhesion, signal transduction, immune response regulation, inflammatory pathways, and the intricate formation of the tumor microenvironment. While preliminary studies have begun to elucidate the phenotypic diversity and bioinformatic characteristics of Integrin α L across various diseases, there remains a paucity of comprehensive reviews addressing the functional roles and underlying mechanisms of Integrin α L in different pathological contexts. This review aims to delineate the fundamental structure and function of Integrin α L, while also summarizing the relationship between its expression patterns and functional attributes with respect to the invasive potential, metastatic capabilities, immune evasion strategies, and clinical outcomes of tumor cells and patients across a spectrum of tumor types. Furthermore, we highlight the significant involvement of Integrin α L in non-tumor-related diseases, including atherosclerosis, systemic sclerosis, depression, and rheumatoid arthritis. Additionally, we assess the potential of Integrin α L as a molecular biomarker for the diagnosis of specific diseases and tumors, which may pave the way for novel therapeutic targets in the treatment of associated conditions and malignancies.

## Introduction

**Figure 1:**
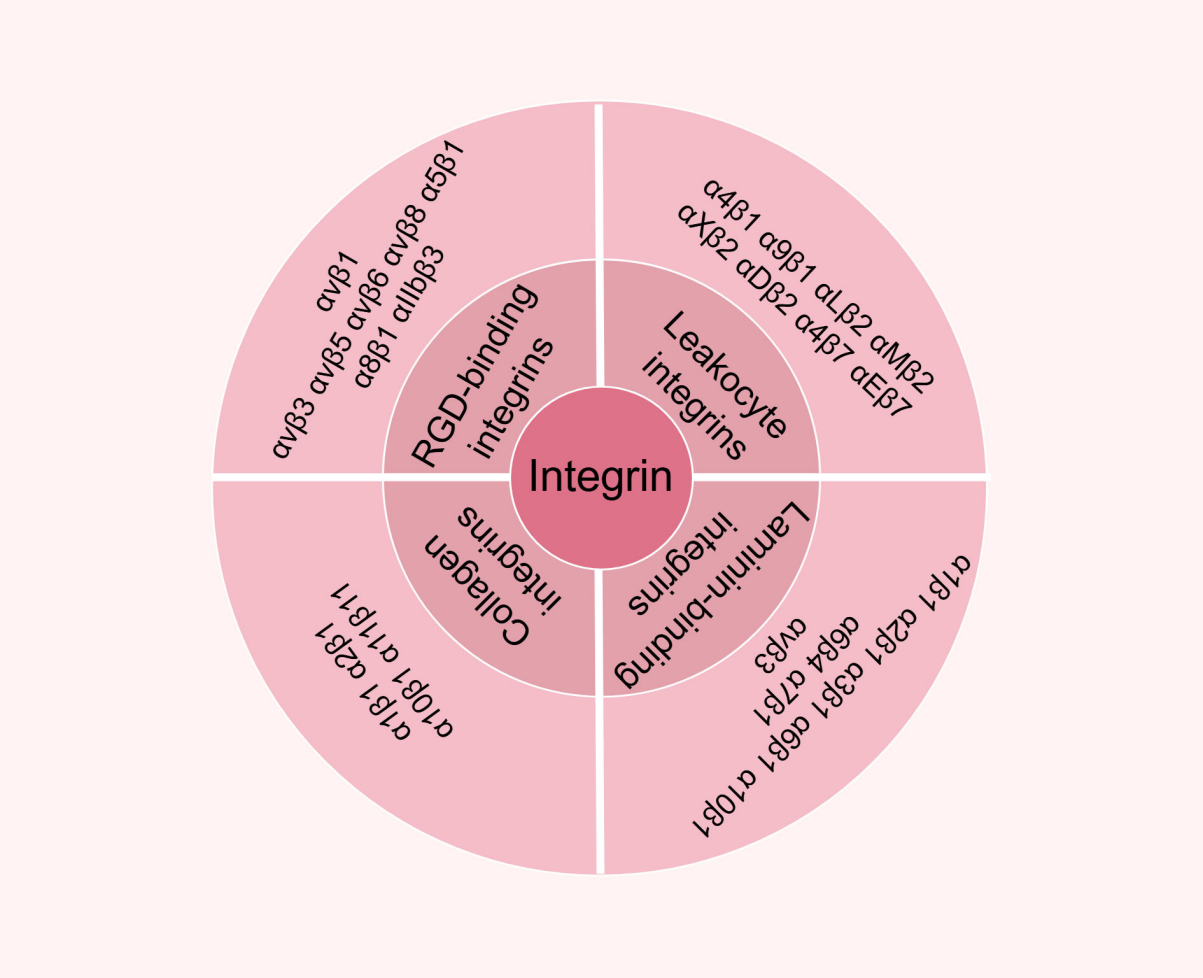
Basic information and structure of Integrin α L. Based on the type of ligand bound by integrins, they can be broadly classified into four categories: the first category is laminin-binding integrins, including α1β1, α2β1, α3β1, α6β1, α7β1, and α6β4; the second category is collagen-binding integrins covering α1β1, α2β1, α3β1, α10β1, and α11β1; the third category is leukocyte-binding integrins, specifically αLβ2, αMβ2, αXβ2, and αDβ2; and the fourth category is integrins recognising RGD sequences, including α5β1, αVβ1, αVβ5, αVβ6, αVβ8, and αIIbβ3.

Integrins, which are significant constituents of the immunoglobulin superfamily, represent a class of heterodimeric transmembrane proteins composed of two subunits, α and β. These proteins serve as critical cell surface receptors. To date, approximately 20 distinct integrin molecules have been identified, each exhibiting unique and varied functional characteristics. Integrins can be categorized into four groups based on the specific ligands they bind. The first group includes laminin-binding integrins, such as α1β1, α2β1, α3β1, α6β1, α7β1, and α6β4. The second category comprises collagen-binding integrins, including α1β1, α2β1, α3β1, α10β1 and α11β1. The third group encompasses leukocyte-binding integrins, including αLβ2, αMβ2, αXβ2, and αDβ2. The fourth group comprises integrins that recognize RGD (Arg-Gly-Asp) sequences, such as α5β1, αVβ1, αVβ5, αVβ6, αVβ8, and αIIbβ3 (1) (**Figure 1**). Integrins are integral to the cellular response to a variety of signals, primarily due to their ability to accurately detect and respond to numerous stimuli within the tumor microenvironment. The interactions between integrins and the Extra-Cellular Matrix (ECM) provide the essential impetus for cell migration and invasion. Additionally, integrins engage with cytoskeletal proteins through specific structural domains, thereby creating a transmembrane connection between the ECM and the cytoskeleton, which facilitates bidirectional transmembrane signal transduction. This integration of signaling is vital for the regulation of key processes, including intercellular adhesion, migration, proliferation, and differentiation (2).

Among the various integrins specific to leukocytes, Integrin α L, a crucial component of LFA-1, is integral to the mechanisms of leukocyte adhesion and migration. This review aims to elucidate the fundamental structure and function of Integrin α L, while also summarizing the relationship between its expression patterns and functional characteristics, particularly in relation to the invasive potential and metastatic capabilities of tumor cells, immune evasion strategies, and the clinical prognosis of patients. Additionally, the review will highlight the significant role of Integrin α L in non-tumor-related diseases, including atherosclerosis, systemic sclerosis, depression, and rheumatoid arthritis. Building on this foundation, the feasibility of utilizing Integrin α L as a molecular marker for the diagnosis of tumors and certain immune-mediated diseases will be evaluated. Lastly, as a prototypical transmembrane protein, there is an expectation to identify or synthesize specific small molecule inhibitors for the treatment of clinically relevant conditions.

## 1 Structure and function of Integrin α L

### 1.1 Structure

**Figure 2:**
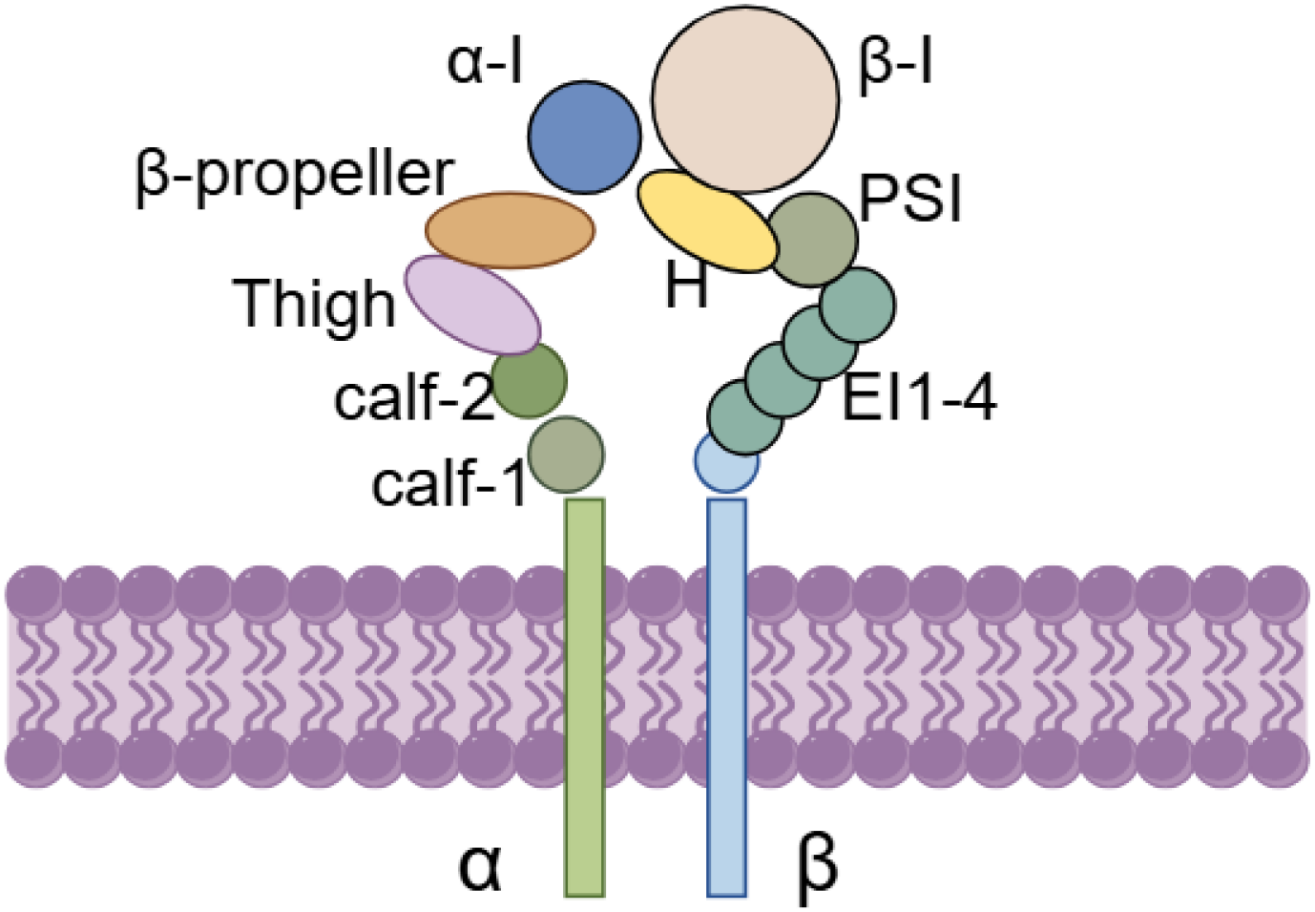
Structure of Integrin α L and LFA-1. Integrin α L (also known as CD11α), is a transmembrane protein encoded by the Integrin α L gene. It belongs to the family of structural domain I containing α integrins and binds to the β 2 chain (Integrin subunit Beta 2, ITGB2), which together form the integrin lymphocyte function-associated antigen-1 (LFA-1). The extracellular terminus of Integrin α L protein contains α-I, β-propeller, Thigh, Calf-2 and Calf-1 domains. The extracellular terminus of ITGB2 protein contains β-I, PSI, H, EI1, EI2, EI3 and EI4 doamins.

**Figure 3:**
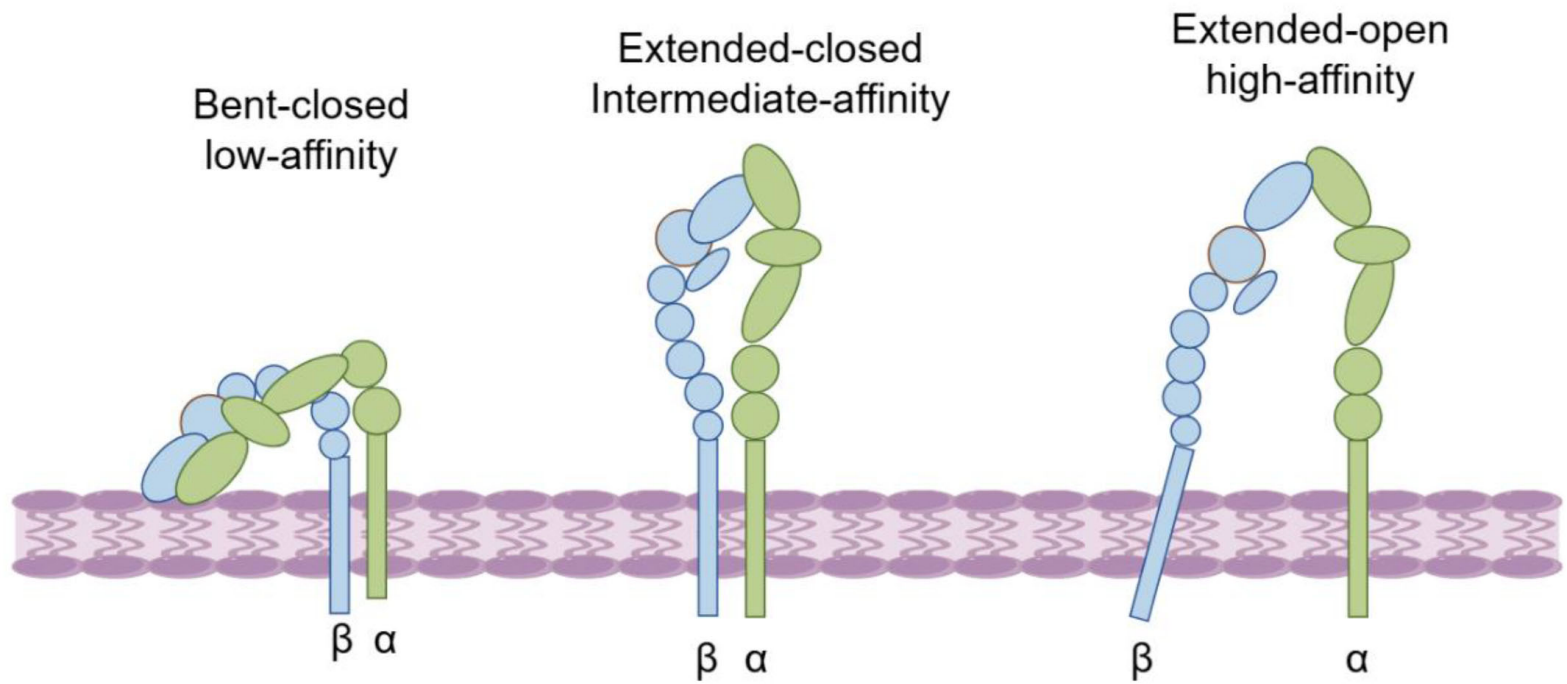
Integrins exhibit three core conformational states: inactive (curved and closed), transitional (partially expanded but still closed) and active (fully expanded and open).

Integrin alpha L, commonly referred to as CD11a, is a transmembrane protein encoded by the Integrin α L gene, which is situated on human chromosome 16p11.2. This protein is classified within the family of α integrins that contain structural domain I and associates with the β2 chain (integrin subunit beta 2, ITGB2) to form the integrin known as LFA-1. LFA-1 is a significant component of the leukocyte adhesion molecule integrin family (**Figure 2**). The Integrin α L protein features a substantial extracellular structural domain that encompasses critical ligand binding sites, which are essential for the regulatory mechanisms governing integrin function. The ability of integrins to bind to ligands is often tightly regulated through dynamic changes in their conformation and aggregation state. Integrins can exist in three primary conformational states: an inactive state (characterized as curved and closed), a transitional state (partially unfolded yet still closed), and an active state (fully unfolded and open) (**Figure 3**). The activation of integrins is generally facilitated by cell surface receptors, including chemokine receptors and T-cell receptors. Importantly, the upper region of the α-subunit, known as the I-domain, plays a crucial role in ligand binding. Upon ligand interaction, the I-domain undergoes a conformational shift to achieve an active state, which subsequently triggers an “outward to inward” signaling cascade. These biochemical and mechanical signals are transmitted to the cell’s interior, where they meticulously regulate a variety of cellular functions.

### 1.2 Function

Integrin α L is expressed across various leukocyte types, including T cells, B cells, macrophages, and neutrophils (3, 4). From a biochemical perspective, Integrin α L mediates four principal functional domains (5): intercellular adhesion (6), signal transduction (7), immune response modulation, and (8) disease pathogenesis.

As a transmembrane glycoprotein, Integrin α L features an N-terminal extracellular domain that exhibits specific binding affinity for multiple ligands, particularly members of the intercellular adhesion molecule family (ICAM-1 to ICAM-3). This molecular interaction facilitates the formation of stable mechanical connections with either extracellular matrix components or adjacent cellular surfaces, thereby supporting critical immunological processes including immune cell adhesion, directed migration, and immunological synapse formation (9–11). The Integrin α L and ICAM 1–3 engagement serves as the structural foundation for immune cell infiltration and effector functions within inflammatory microenvironments (8, 12).

Regarding signal transduction mechanisms, conformational activation of Integrin α L initiates intracellular signaling cascades that regulate fundamental cellular processes such as proliferation, migration, and apoptosis (9, 13). Functioning as a prototypical bidirectional signaling receptor, Integrin α L mediates both “outside-in” signaling (transducing extracellular cues to intracellular effectors) and “inside-out” signaling (modulating extracellular ligand affinity in response to intracellular stimuli). The dynamic equilibrium between active and inactive conformational states enables precise regulation of immune cell adhesive strength and effector functions (14–16). The αL subunit (CD11a) plays a determinant role in ligand specificity and signaling properties, establishing its central position within intercellular communication networks and signaling pathways.

**Figure 4:**
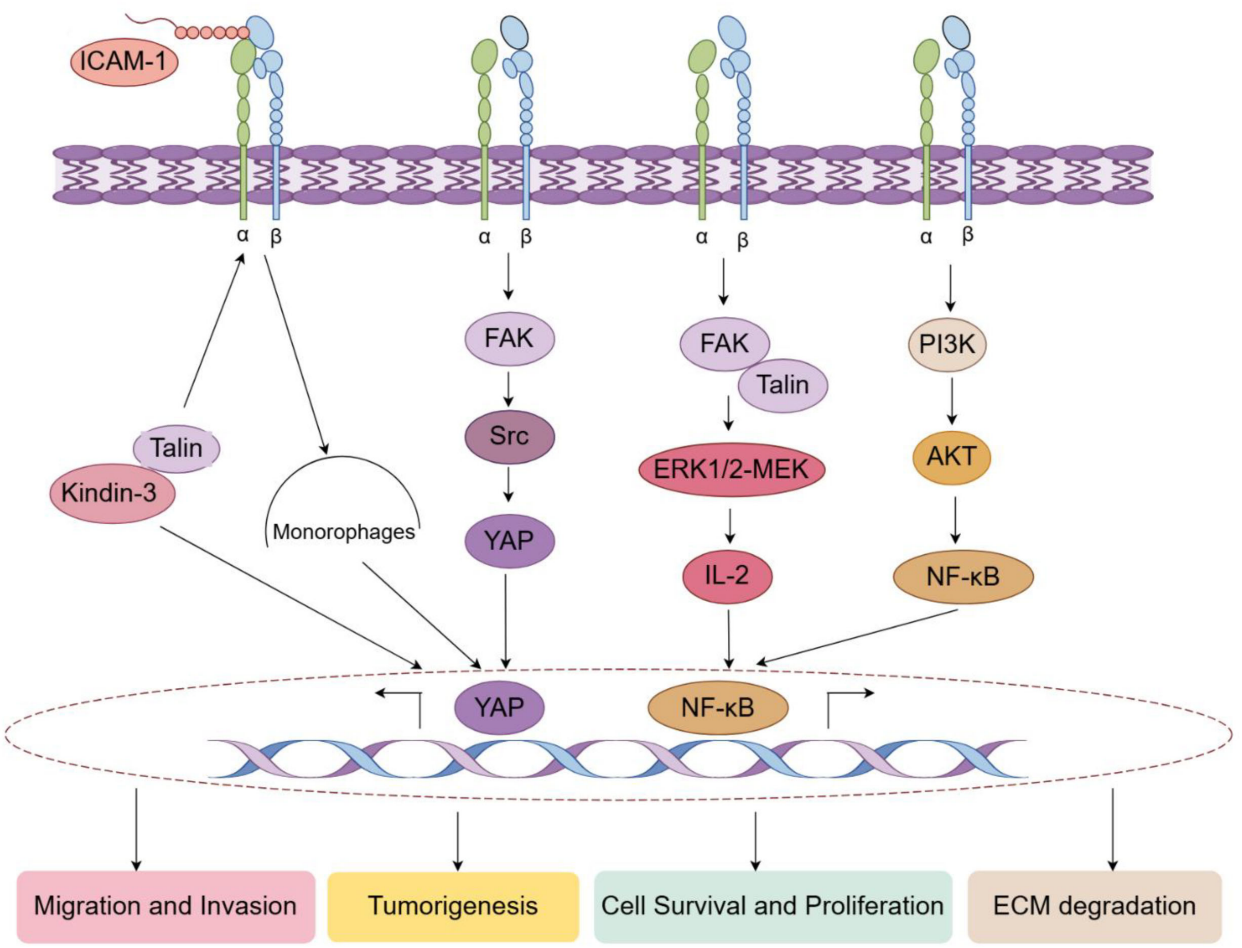
Molecular mechanisms by which Integrin α L affects tumorigenesis, cell proliferation, cell migration and ECM degradation.

Furthermore, Integrin α L plays a pivotal role in immune response execution (17, 18). Upon successful recognition of MHC-antigen complexes on antigen-presenting cells, Integrin α L on the T cell surface is rapidly activated and specifically binds to ICAM-1 on the antigen-presenting cell. This sustained intercellular contact is essential for T cell proliferation. Moreover, the synergistic interaction of Integrin α L with CD3 and CD28 is significantly involved in the signaling pathway, effectively lowering the activation threshold of T cells, which subsequently initiates the phosphorylation of extracellular signal-regulated kinase 1/2 (ERK1/2) and enhances the production of interleukin-2 (IL-2) (19) (**Figure 4**). Consequently, it can be inferred that Integrin α L -mediated immune synapse formation is a critical process in the cytotoxic activity of T cells and natural killer (NK) cells against target cells.

Additionally, Integrin α L exhibits significant functions in cancer biology. It participates in angiogenesis and contributes to cancer initiation and progression (7, 20–23), while also modulating immune responses, inflammatory processes, and tumor microenvironment composition, thereby influencing tumor growth, invasion, and metastatic potential (24, 25, J. 26). This characteristic has allowed Integrin α L to assume diverse roles in the pathogenesis of various malignancies, including renal cancer, colorectal cancer, melanoma, and squamous cell carcinoma of the head and neck (27–30). Ongoing investigations into different tumor types have underscored the complexity and significance of its function.

## 2 Function of Integrin α L in lymphocytes

### 2.1 Role of Integrin α L in T cells

LFA-1, a critical adhesion molecule in T cells, is integral to the immune response, with its primary component being Integrin α L. Initial research, notably by Krensky et al. in 1985 (31), established that inherited defects in Integrin α L lead to dysfunctions in cytotoxicity and proliferation, highlighting the essential role of Integrin α L in T cell functionality. However, the specific functions and regulatory mechanisms of Integrin α L were not fully understood at that time. To elucidate the role of Integrin α L, Shimizu et al. conducted a study in 1992 (32) that investigated the impact of cross-linking T cell-specific helper molecules CD7 and CD28 on T cell adhesion properties. Their findings indicated that the cross-linking of CD7 and CD28 modulates T cell adhesion behavior and demonstrated that Integrin α L and CD2 serve distinct functions in this process.

Advancing the research, Anikeeva et al. in 2005 examined the role of Integrin α L in the cytolytic activity of Cytotoxic T Lymphocytes (CTL) (5). They concluded that while alternative adhesion mechanisms can initiate the degranulation of CTLs, effective binding of Integrin α L to ICAM-1 is crucial for the successful elimination of target cells via released granules. Additionally, Integrin α L binding promotes the formation of a larger contact area known as the peripheral Supra-Molecular Activation Cluster (pSMAC), facilitating the proximity of granules and Golgi complexes to the interface. Their data suggest that Integrin α L conveys a unique signal essential for accurately directing released particles toward the surface of antigen-presenting target cells, thereby ensuring their effective destruction. In a more recent investigation, McNamara et al. (2017) (33) explored the potential role of Integrin α L in the formation and maintenance of T cell memory. Their research revealed that the up-regulation of Integrin α L allows liver-resident memory T cells to effectively patrol and stabilize within the hepatic sinusoidal environment. This finding contrasts with earlier studies that indicated genetic defects in Integrin α L result in abnormal T cell function, further underscoring the importance of Integrin α L in T cell memory dynamics.

Beyond its structural role in immunological synapse formation and vesicular transport, emerging evidence demonstrates that Integrin α L precisely regulates T cell activation through distinct intracellular signaling pathways. Upon ICAM-1 engagement, Integrin α L initiates outside-in signaling that facilitates its association with focal adhesion kinase (FAK), inducing phosphorylation at Y397 and subsequent activation of Src family kinases (34, 11). Consistent with this mechanism, Integrin α L inhibition significantly reduces FAK-dependent phosphorylation and impairs T cell activation (14). These findings establish the Integrin α L -FAK-Src axis and related transduction pathways (YAP, Yes Associated Protein) as critical regulators of T cell responses (**Figure 4**). Nonetheless, despite these advancements, the comprehensive functions and regulatory mechanisms of Integrin α L warrant further in-depth exploration by researchers.

### 2.2 Role of Integrin α L in leucocyte

Integrin α L, a key integrin found on leukocytes, interacts with ITGB2 to form the LFA-1 complex, which is essential for the adhesion and migration of leukocytes. Research conducted by Laudanna et al. (2002) identified two distinct mechanisms through which chemokines rapidly activate leukocyte integrins (35). These mechanisms serve different functions in the processes of leukocyte adhesion and migration; the high-affinity state is crucial for leukocyte adhesion, while lateral movement enables the swift transit of leukocytes through the cardiovascular system. This suggests that YAP may modulate leukocyte adhesion and migration by influencing the expression levels of Integrin α L and ITGB2. Additionally, a significant study by Liu et al. (2018) (36) demonstrated that YAP can substantially enhance the expression of Integrin α L and ITGB2 in tumor cells, facilitated by PR domain zinc finger protein 4 (PRDM4), an important regulatory molecule. This discovery not only uncovers a novel role for YAP in tumor invasion but also clarifies the mechanism by which Integrin α L and ITGB2 contribute to the invasive behavior of tumor cells, specifically by enabling their traversal across the endothelial cell layer. This insight offers a new framework for understanding the process of tumor metastasis.

### 2.3 Binding analysis of Integrin α L and ICAMs

A considerable portion of the functions of Integrin α L is fundamentally associated with its biological effects, which are mediated through its interaction with ICAMs and represent a critical component of LFA-1. Lefort et al. elucidated the mechanism underlying the interaction between Integrin α L and ICAMs during the recruitment of neutrophils to inflamed tissues (37). Their observations indicated that the “inside-out” activation mode of Integrin α L, characterized by the activation of intracellular signaling pathways, leads to a significant conformational change from an inactive to an expanded state. This conformational shift facilitates the binding of Integrin α L to ICAMs, thereby affecting leukocyte adhesion dynamics. However, the complete activation of Integrin α L relies on an “outside-in” activation mode, which is initiated by ligand binding. Additionally, further mechanical stimulation is required to enhance affinity maturation. This finding lays a solid foundation for a comprehensive exploration of the activation mechanisms of Integrin α L. In a prior investigation, Sadhu and colleagues focused on the key amino acid residues within the LFA-1 I structural domain (Integrin α L) that interact with ICAM-3 (38). Their research revealed that the Ile-Lys-Gly-Asn sequence, located at the N-terminal end of the LFA-1 I structural domain, is critical for binding to ICAM-3, although it is less significant for binding to ICAM-1. Moreover, it was shown that the aspartic acid residues Asn-129 and Asp-137 are also essential for ICAM-3 binding. These findings further substantiate the existence of distinct functional subfamilies for specific ligand binding within the LFA-1 I structural domain (Integrin α L), which are vital for the proper functioning of leukocytes and the precise regulation of the immune response.

Building on this foundation, the molecular mechanism underlying the synergistic interaction of integrin α L with Talin and kindlin further clarifies the coupling between intracellular signaling and extracellular adhesion. Integrin α L pairs with the β2 subunit (CD18) to form the heterodimeric receptor LFA-1. Talin binding to the intracellular domain of the β2 subunit induces a conformational transition of the integrin from a low-affinity to a high-affinity “stretched” state. Subsequently, kindlin-3 binds to the membrane-proximal region of the β2 subunit via its Pleckstrin Homology (PH) domain, further stabilizing the high-affinity conformation and promoting ligand binding (6, 39). Notably, conserved threonine residues (Thr788/Thr789) within the cytoplasmic domain of the β2 subunit function as a phosphorylation switch, where phosphorylation status determines preferential binding of Talin versus kindlin to finely regulate integrin α L activity (40). These molecular events elucidate the kinetic basis of integrin affinity modulation and provide a mechanistic rationale for integrin α L’s functional plasticity across diverse cellular microenvironments (**Figure 4**).

Furthermore, Integrin α L and ICAMs engagement triggers “outside-in” signaling, inducing FAK phosphorylation (11, 14). Activated FAK then mediates phosphatidyqinositol‐3 kinase(PI3K)-protein kinase B(Akt) pathway activation, establishing a FAK–PI3K–AKT signaling axis (41, 42). Consistent with the canonical mechanism, PI3K-generated phosphatidylinositol-trisphosphate (PIP_3_) recruits both PDK1 and AKT to the plasma membrane via their PH domains. This recruitment enables PDK1 to phosphorylate AKT at Thr308, activating AKT (43). Collectively, this evidence suggests Integrin α L mediated activation of the FAK–PI3K–AKT axis represents a functional link integrating membrane adhesion events with NF-κB dependent transcriptional responses. Together with the Integrin α L–FAK–Src–YAP axis and Talin/Kindlin-mediated activation mechanisms, the FAK–PI3K–AKT–NF-κB axis contributes to a complementary molecular network governing integrin α L regulated adhesion, mechanotransduction, and immune function (**Figure 4**).

### 2.4 Other functions mediated by Integrin α L

The role of ICAM-3 extends beyond leukocytes, demonstrating significant biological effects on various cell types. Research conducted by Kristóf et al. highlighted the critical involvement of ICAM-3 and Integrin α L in the phagocytosis of apoptotic neutrophils by human macrophages (16). The study revealed that the expression levels of both ICAM-3 and Integrin α L were markedly upregulated following the phagocytosis of apoptotic cells by macrophages. Additionally, the necessity of ICAM-3 and Integrin α L in this phagocytic process was substantiated through gene silencing and antibody blockade experiments. The outcomes of these blocking experiments, in conjunction with immunofluorescence staining and spotlight microscopy observations, indicated that ICAM-3 and Integrin α L aggregate at the sites of phagocytosis and co-localize on the macrophage surface, thereby reinforcing their role as phagocytic receptors. Furthermore, it was observed that the phagocytosis of apoptotic neutrophils inhibited the release of inflammatory factors from macrophages, while the blockade of ICAM-3 or Integrin α L partially diminished this inhibitory effect. This finding implies that ICAM-3 and Integrin α L are not only integral to macrophage phagocytosis but may also facilitate the transmission of anti-inflammatory signals from apoptotic neutrophils to macrophages. This mechanism offers new insights into the processes of inflammation resolution and immune homeostasis, as well as potential targets for the development of innovative therapeutic strategies aimed at treating inflammatory and autoimmune disorders (**Figure 4**).

## 3 Function of Integrin α L in tumors

### 3.1 Integrin α L and breast cancer

Breast cancer, recognized as the predominant cause of mortality among female malignancies, has garnered extensive global attention due to its high incidence and fatality rates. Early investigations have elucidated the critical roles of immune cells and the extracellular matrix within the tumor microenvironment in facilitating tumor progression and metastasis. Subsequently, Sökeland et al. (44) delved into the mechanisms of integrins role in tumor cell metastasis in their study and specifically mentioned that Integrin α L may help tumor cells to firmly adhere to vascular endothelial cells by interacting with other cell adhesion molecules, thus significantly facilitating the process of tumor metastasis. This study not only reveals the important role of integrins in tumor metastasis, but also implies that the expression and functional status of Integrin α L, as the α-subunit of LFA-1, may be closely related to the malignant phenotype and metastatic potential of breast cancer. Further mechanistic studies support a critical role for intracellular signaling modules in regulating Integrin α L activity in tumor cells. For example, in breast cancer cells, PDE8A forms a complex with Raf-1 at the plasma membrane, generating a local microdomain with reduced cAMP levels. This attenuates PKA-mediated inhibition of Raf-1, thereby enhancing Raf-1/MAPK signaling and the activity of downstream small GTPases. The resulting signaling cascade promotes inside-out activation and membrane clustering of Integrin α L, significantly increasing tumor cell adhesion to ICAM-1 under physiological shear stress. This facilitates firm adhesion to the vascular endothelium, transendothelial migration, and subsequent metastatic dissemination. Conversely, pharmacological inhibition of PDE8A restores cAMP/PKA-dependent suppression of Raf-1, diminishes Integrin α L function, and thereby reduces adhesion and metastasis in breast cancer cells (45). In 2020, Rojas and his team (46) conducted an in-depth analysis of the relationship between integrin gene expression and patient survival utilizing the Kaplan-Meier Plotter and Molecular Taxonomy of Breast Cancer International Consortium (METABRIC) datasets, and assessed the association between integrin genes and tumor immune cell infiltration in conjunction with the Tumor Immune Estimation Resource (TIMER) platform. They found that the expression level of the Integrin α L gene was significantly associated with the degree of immune cell infiltration in basal-like and HER2^+^ breast cancers, which may predict a better prognosis for patients. However, these studies also suggest that there is often a strong association between high expression of the Integrin α L integrin gene in breast cancer tumor cells and enhanced invasive of tumor cells, increased propensity to metastasis, and deterioration of patient prognosis. Thus, Integrin α L integrins significantly enhance the invasive and migratory ability of breast cancer cells by finely modulating the interaction between tumor cells and extracellular matrix, thereby accelerating the process of tumor growth and spread.

### 3.2 Integrin α L and colon cancer

The high incidence and mortality associated with colon cancer have consistently raised significant concerns within the medical community. The pathogenesis of this malignancy is intricate and multifactorial. Research has identified Integrin α L integrins as pivotal in the study of colon cancer, with their mechanisms of action encompassing three-dimensional growth, genetic associations, intercellular communication, and exosome-mediated processes.

Valcárcel and his team (47) constructed a three-dimensional cell spheroid model to simulate the growth environment of colon cancer cells. They found that under three-dimensional growth conditions, colon cancer cells significantly increased the expression level of Integrin α L and promoted the secretion of Vascular Endothelial Growth Factor (VEGF) through interaction with soluble ICAM-1, which in turn activated the angiogenesis process and accelerated the formation of hepatic micro-metastases. It was further demonstrated that three-dimensional grown colon cancer cell spheroids had a stronger pro-angiogenesis ability, and Integrin α L played a key role in this process.

In addition, Lange et al.’s group (2023) (48) found a significant association between several integrin genes, including the Integrin α L gene encoding Integrin α L, and Inflammatory Bowel Disease (IBD) through a Genome-Wide Association Study (GWAS). IBD is a common inflammatory disease of the bowel and one of the preexisting lesions of colorectal cancer. Through eQTL analysis, IBD risk alleles were found to upward the expression levels of integrin genes, which further suggests that Integrin α L may be involved in the complex link between intestinal inflammation and cancer.

In 203, Guo et al. (49) found that ITGB3^+^ and ITGAM^+^ exosome sub-populations were closely associated with colon cancer progression using single exosome analysis. Among them, ITGB3+ exosome were enriched in Integrin α L, which could promote the proliferation, migration and invasion of tumor cells; whereas ITGAM^+^ exosome exhibited inhibition of tumor progression and may originate from macrophages. It was revealed that Integrin α L may regulate the progression process of colon cancer by affecting exosome mediated intercellular communication, providing a new potential target for colon cancer treatment.

In conclusion, Integrin α L plays a multifaceted role in colon cancer, including the promotion of tumor angiogenesis, participation in tumor metastasis, influence on T cell function and intercellular communication through exosome mediated communication. These findings not only enhance our comprehension of the pathogenesis of colon cancer but also offer novel concepts and strategies for the treatment of colon cancer.

### 3.3 Integrin α L and lung cancer

Lung cancer is the most common type of cancer globally, characterized by the highest rates of incidence and mortality, which poses a considerable challenge to public health on an international scale. As research into targeted therapies progresses, Integrin α L integrins have emerged as a prominent area of investigation. A previous study conducted by Wang et al. (24) utilized data from the The Cancer Genome Atlas (TCGA) database to demonstrate that the expression levels of Integrin α L in Lung Adenocarcinoma (LUAD) tissues were significantly lower than those in normal tissues. This reduced expression was found to correlate closely with poor patient prognosis. The authors subsequently developed a prognostic prediction model, termed the 3-ITG model, which incorporated Integrin α L and was shown to enhance the accuracy of prognosis predictions for LUAD patients when integrated with pathological staging. Biological pathway analyses indicated that the high-risk group exhibited significant enrichment in multiple pathways associated with metastasis, suggesting that the 3-ITG model may contribute to the adverse prognosis of LUAD patients by facilitating metastasis and immune evasion. However, the study did not elucidate the specific mechanisms underlying the role of Integrin α L in these processes.

In a subsequent investigation, the same research team conducted a comprehensive analysis of Integrin α L expression in Non-Small Cell Lung Cancer (NSCLC) utilizing TCGA and Gene Expression Omnibus (GEO) databases (50). Their findings revealed that Integrin α L expression levels in NSCLC tissues were similarly diminished compared to normal tissues, with low expression being significantly associated with poor patient outcomes. Further analysis indicated a positive correlation between Integrin α L expression and various immune cell types and immune-related factors, suggesting that Integrin α L may play a role in modulating the tumor immune microenvironment. Additionally, Integrin α L expression was associated with immune checkpoint-related molecules, providing further evidence of its potential involvement in immune evasion in lung cancer. Nonetheless, the precise mechanisms by which Integrin α L interacts with these immune cells and factors remain to be clarified.

To further validate the mechanisms of Integrin α L in lung cancer and find potential therapeutic targets, Lin and his team (51) constructed an expression panel comprising 12 genes (TxflSig) and 7 genes (TxflSig1) and conducted in-depth studies on the genes closely associated with immunosuppressive activity. Their discovered that Integrin α L, a recently identified biomarker of lung cancer response to Immune Checkpoint Blockade (ICB) therapy, demonstrated enhanced robustness and predictive value compared to ICB biomarkers currently employed in NSCLC, such as PD-L1. The discovery of this new finding provides potential targets and strategies for immunotherapy in lung cancer. Furthermore, this study has also confirmed that Integrin α L may serve as a biomarker for assessing the immune micro-environment in NSCLC patients. However, the specific mechanism of Integrin α L with immune checkpoints remains unclear.

To thoroughly examine the specific mechanisms underlying the action of Integrin α L in lung cancer and its association with immune checkpoints, Zhang et al. (52) conducted additional investigations utilizing the TCGA database and single-cell sequencing analysis. They found that the expression level of Integrin α L in NSCLC patients was positively correlated with a variety of immune cell types and immune-related factors, which further confirmed that Integrin α L may be involved in the regulation of the tumor immune microenvironment. Meanwhile, they also found that the expression of Integrin α L was significantly associated with immune checkpoint molecules, suggesting that Integrin α L may play an important role in the immune escape mechanism of lung cancer. In addition, this study revealed that lncRNA PCBP1-AS1 may affect Integrin α L expression by regulating the expression levels of miR-9-5p and miR-424-5, which in turn has a profound impact on the immune micro-environment of lung cancer and patient prognosis (50). This finding provides new explanations and ideas for the specific mechanism of action in the Integrin α L 3-ITG model and the specific mechanism of action of immune checkpoints.

### 3.4 Integrin α L and gastric cancer

Gastric cancer, recognized as one of the most prevalent and fatal malignancies globally, is characterized by subtle symptoms in its early stages. This insidious nature complicates treatment efforts and significantly diminishes patient survival rates. As investigations into gastric cancer have advanced, the role of Integrin α L has emerged as a critical area of focus within this research domain. The expression levels of Integrin α L are intricately linked to the modulation of the tumor microenvironment, patient prognosis, and the extent of tumor cell infiltration.

Zhou et al. (53) pioneered an in-depth analysis using databases such as TIMER, Gene Expression Profiling Interactive Analysis (GEPIA) and TISIDB (an integrated repository portal for tumor-immune system interactions). The researchers observed a significant upward of Integrin α L expression in gastric cancer tissues relative to normal tissues. Moreover, this expression level demonstrated a positive correlation with the degree of infiltration of various immune cell sub-populations, including CD8^+^ T cells, CD4^+^ T cells, B cells, macrophages, and neutrophils. This finding demonstrates the significant regulatory function of Integrin α L in the gastric cancer micro-environment.

Furthermore, Zhou et al. (53) demonstrated that the expression level of Integrin α L was closely correlated with that of a number of immune modulators, including PD-L1, CTLA4 and LAG3. This finding further supports the hypothesis that Integrin α L may influence gastric cancer progression by regulating the tumor immune micro-environment. To test this hypothesis, they performed immunohistochemical staining and found that the expression of Integrin α L in gastric cancer tissues showed a positive correlation with the expression level of PD1 as well. This result suggests that Integrin α L may accelerate the progression of gastric cancer by promoting PD1 expression to enhance the immune escape ability of tumor cells.

In summary, the upward of Integrin α L expression in gastric cancer is closely associated with poor prognosis. It may accelerate the progression of gastric cancer by regulating the immune micro-environment and promoting immune escape. Therefore, Integrin α L is expected to be a new target for gastric cancer treatment.

### 3.5 Integrin α L and leukemia

Leukemia, commonly referred to as a blood cancer, has been a significant concern within the medical community, particularly with regard to the challenges associated with its treatment, despite its relatively low prevalence compared to other forms of cancer. In recent years, important progress has been made in research on Acute Myeloid Leukemia (AML). Hu et al. (54) conducted an in-depth analysis of RNA sequencing data from AML patients in the TCGA-LAML database by bioinformatics means and combined it with clinical information. The researchers discovered that Integrin α L exhibited a markedly elevated expression status in AML patients, which was found to be significantly associated with a less favorable treatment prognosis. Additional experimental findings demonstrated that Integrin α L knockdown markedly suppressed AML cell proliferation and induced apoptosis, while concurrently reducing the number of CD45^+^ cells, decreasing spleen weight, and extending the survival duration of mice in the AML mouse model. These findings suggest that Integrin α L may facilitate AML progression by modulating cytokine production and influencing the quantity of Myeloid-Derived Suppressor Cells (MDSC). However, the authors did not explore the specific mechanisms through which Integrin α L contributes to adverse prognostic outcomes. In a separate investigation, Li et al. (55) examined the adhesion signaling pathway of AML cells from a proteomics standpoint, operating under the assumption that Integrin α L could serve as a potential prognostic biomarker for AML patients. They used Reverse Phase Protein Array (RPPA) technology to examine the expression levels of multiple adhesion-related proteins in AML patient samples and combined this with bioinformatics analysis methods to classify patients into different states of adhesion signaling pathway activation. The Integrin α L state was identified as an “intermediate activation” state, linked to FLT3 and NPM1 mutations, gender, and response status. The results of this study provide further evidence that Integrin α L is a prognostic biomarker for AML patients. Therefore, Integrin α L may contribute to cancer progression by influencing the immune micro-environment in AML and play a pivotal role in the development of leukemia. Consequently, Integrin α L may become a crucial target for AML diagnosis, prognostic determination and therapeutic strategies.

### 3.6 Integrin α L and melanoma

Melanoma, a highly aggressive neoplasm derived from melanocytes, has garnered significant attention within the oncology community due to its pronounced malignancy and the complexities associated with late-stage treatment. Recent statistical analyses indicate a global increase in melanoma incidence, underscoring the necessity for comprehensive investigations into its pathogenesis. To better understand the mechanisms contributing to the poor survival rates in metastatic melanoma, Ji and colleagues (56) performed the identification of differential expressed genes. They identified 464 Differential Expressed Genes (DEGs) between primary and metastatic tumor tissues, among which Integrin α L plays a crucial role in regulating the prognosis of metastatic melanoma patients. This finding reveals a potential role for Integrin α L in melanoma progression. Conversely, Kwak et al. (57) focused their research on the ICH gene. Their findings indicated that this gene is significantly linked to a generalized increase in numerous immune cell subset. Among these ICH genes, Integrin α L is of particular interest as it significantly influences the expression of immune activation and effector function genes. While the study did not test the hypothesis that there is a unique correlation between each immune cell subset and a specific ICH gene, a significant correlation was found between Integrin α L and immune cell subset. Subsequently, Deng led a team (25) to demonstrated t the critical role of Integrin α L in melanoma. They identified higher expression of Integrin α L in melanoma tissues than in normal tissues in a large amount of data and further verified that there was a significant correlation between Integrin α L expression and the infiltration of multiple immune cells. These findings indicate that Integrin α L may be a key regulator of the immune response and tumor micro-environment in melanoma. However, Niu and colleagues (58) demonstrated via animal studies that Integrin α L knockout significantly inhibited tumor growth, concomitant with a decrease in the number of regulatory T cells (Treg cells) in the spleen, peripheral blood, and mesenteric lymph nodes. Furthermore, treatment of Integrin α L ^+^/^+^ tumor-bearing mice with BIRT377(an Integrin α L inhibitor)elicited comparable effects, specifically the suppression of tumor growth and a reduction in Treg cell numbers. Additionally, analyses using the TIMER and GEPIA databases further showed that Integrin α L expression was positively correlated with both the expression of *Foxp3* (a canonical marker of Treg cells) and tumor TNM stage (a key indicator of tumor progression). Notably, this correlation was consistently observed in melanoma and across multiple other tumor types. In summary, the high expression of Integrin α L in melanoma and its close correlation with immune cell infiltration reflect its significant potential as a new biomarker for assessing the prognosis of melanoma patients and as a therapeutic target. With the in-depth study of the mechanism of action of Integrin α L in melanoma, it is expected to provide new ideas and methods for the treatment of melanoma.

### 3.7 Integrin α L and glioma

Gliomas, characterized by their varied growth locations and invasive properties, pose considerable therapeutic challenges, particularly in the case of high-grade gliomas as classified by the World Health Organization (WHO). The high rates of recurrence and mortality associated with these tumors have consistently presented significant obstacles in clinical management. In response to these challenges, targeted therapy has emerged as a crucial therapeutic approach. Nevertheless, the identification of appropriate therapeutic targets remains a contentious and complex issue within glioma research. A recent investigation conducted by Costa and colleagues (59) explored the role of Glioma-Associated Microglia/Macrophages (GAM) in Low-Grade Glioma (LGG). Utilizing advanced methodologies such as immunocytochemistry and RNA-Scope assays, the researchers discovered a markedly elevated expression of the Integrin α L gene in GAM, which was significantly distinct from the expression levels observed in the Disease-Associated Microglia (DAM) population. Notably, a similar increase in Integrin α L expression was also detected in samples from pilocytic astrocytoma. These findings not only underscore the critical involvement of Integrin α L in the biology of LGG but also provide robust experimental support for its regulatory significance within GAM. While the precise mechanisms through which Integrin α L operates in glioma remain to be elucidated, its strong association with immune cell infiltration and its essential role in modulating the immune microenvironment suggest that Integrin α L may serve as a promising target for targeted therapeutic interventions in glioma.

### 3.8 Integrin α L and drugs against cancer

Integrins constitute a critical category of cell adhesion receptors that serve multifaceted roles as signaling molecules, mechanical sensors, and essential components of the cellular migration apparatus. These receptors are involved in nearly every phase of cancer progression, encompassing the development of primary tumors and the subsequent metastatic process. A significant association has been established between integrin functionality and Integrin α L, a connection that has attracted substantial interest from the biotechnology and pharmaceutical sectors, where it has emerged as a promising target for therapeutic intervention. Recent investigations have highlighted the potential of integrin inhibitors as viable therapeutic agents. Liang and his research team (60) utilized a variety of biological experimental methodologies, including RNA sequencing, CyTOF, isolation and culture of CD8+ T cells, and flow cytometry analysis. Their results indicated that the ectopic expression of NPC1L1 in Pancreatic Adenocarcinoma (PAAD) cells inhibited the activation and proliferation of anti-tumor CD8+ T cells within the tumor microenvironment. Furthermore, the overexpression of NPC1L1 facilitated the evasion of immune surveillance by CD8+ T cells, which are critical for the development and progression of PAAD. The findings demonstrated that PAAD cells expressing NPC1L1 function as a checkpoint molecule, thereby inhibiting the cytotoxic activity of anti-tumor CD8+ T cells through the interaction between NPC1L1 and Integrin α L. It can be inferred that the NPC1L1 inhibitor Ezetimibe enhances CD8+ T cell-mediated anti-tumor responses and works synergistically with PD-1 blockers to improve therapeutic efficacy in PAAD treatment. In a prior study, Springer et al. (61) proposed, based on an extensive review of numerous studies, that the micro-clustering of LFA-1 facilitates the formation of intervening LFA-1-free regions, allowing smaller adhesion molecules (e.g., T cell receptors) to interact with one another rather than being inhibited by larger receptors. Consequently, the micro-clustering of LFA-1 may be essential for its multifunctional roles, stabilization of adhesion, and antigen recognition. As research on Integrin α L progresses, there exists the potential for its application as a targeted therapeutic agent in anti-tumor strategies in the future.

### 3.9 The role of Integrin α L in immune evasion and immune checkpoint modulation

Integrin α L mediated immune evasion primarily occurs through its bidirectional signaling, which tightly couples lymphocyte adhesion to effector function. During “inside-out” activation, intracellular signals (such as those from T-cell receptor engagement or chemokines) trigger a conformational shift in Integrin α L from a low-affinity to a high-affinity state, facilitating enhanced ligand binding. This conformational change promotes clustering at the plasma membrane, thereby ensuring stable adhesion of effector T and NK cells to ICAM-1 on endothelial or target cells, and supporting immunological synapse formation (62) (63, 64). Subsequently, Integrin α L–ICAM-1 ligation initiates “outside-in” signaling, which involves cross-talk to other integrins and promotes cytoskeletal remodeling, cellular proliferation, and effector responses. Relevant roles of Integrin α L in human diseases (65–67).

The tumor microenvironment can simultaneously perturb both signaling directions to facilitate immune evasion. Firstly, adenosine A2A receptor signaling, PGE_2_, TGF-β, metabolic competition, and immune checkpoints (e.g., PD-1) can suppress Rap1/Talin activity or elevate cAMP–PKA signaling, thereby impairing “inside-out” activation and leading to unstable immunological synapses and diminished cytotoxicity (68). Conversely, tumors can attenuate or reprogram “outside-in” signaling by downregulating or modifying ICAM-1, releasing soluble ICAM-1, altering ECM stiffness, or redirecting integrin signals to preferentially support immunosuppressive cell subsets. Collectively, these mechanisms block granule polarization and enhance immunosuppressive cell functions (26, 69, 70). Consequently, therapeutic strategies designed to overcome tumor immune evasion should seek to restore “inside-out” activation in T and NK cells, while optimizing the adhesion microenvironment to support “outside-in” signaling. However, such approaches must be precisely targeted to avoid inadvertently augmenting integrin-dependent advantages in tumor cells.

Substantial cross-regulation exists between Integrin α L and immune checkpoints. In tumor cells and tumor-associated stromal cells, Integrin α L activation induces FAK/Src kinase activity, engaging downstream pathways including PI3K/AKT signaling (14). These pathways can upregulate immunosuppressive molecules such as PD-L1, IDO, and TGF-β through transcriptional and epigenetic mechanisms, thereby reinforcing local immune tolerance (71, 72). Additionally, PD-1 on effector T cells engages its ligands and recruit phosphatases like SHP-2, dephosphorylating key tyrosine residues downstream of TCR/CD28 signaling. This inhibits critical pathways, including PLCγ, Vav, and PI3K (73–75), which are essential for Integrin α L inside-out activation and Talin/kindlin recruitment. Consequently, PD-1 signaling not only blocks Integrin α L affinity maturation and clustering but also compromises immunological synapse stability and cytotoxic function, establishing an immunosuppressive network synergistically mediated by integrin and immune checkpoint pathways.

### 3.10 Analysis of Integrin α L expression in different tumors

**Figure 5:**
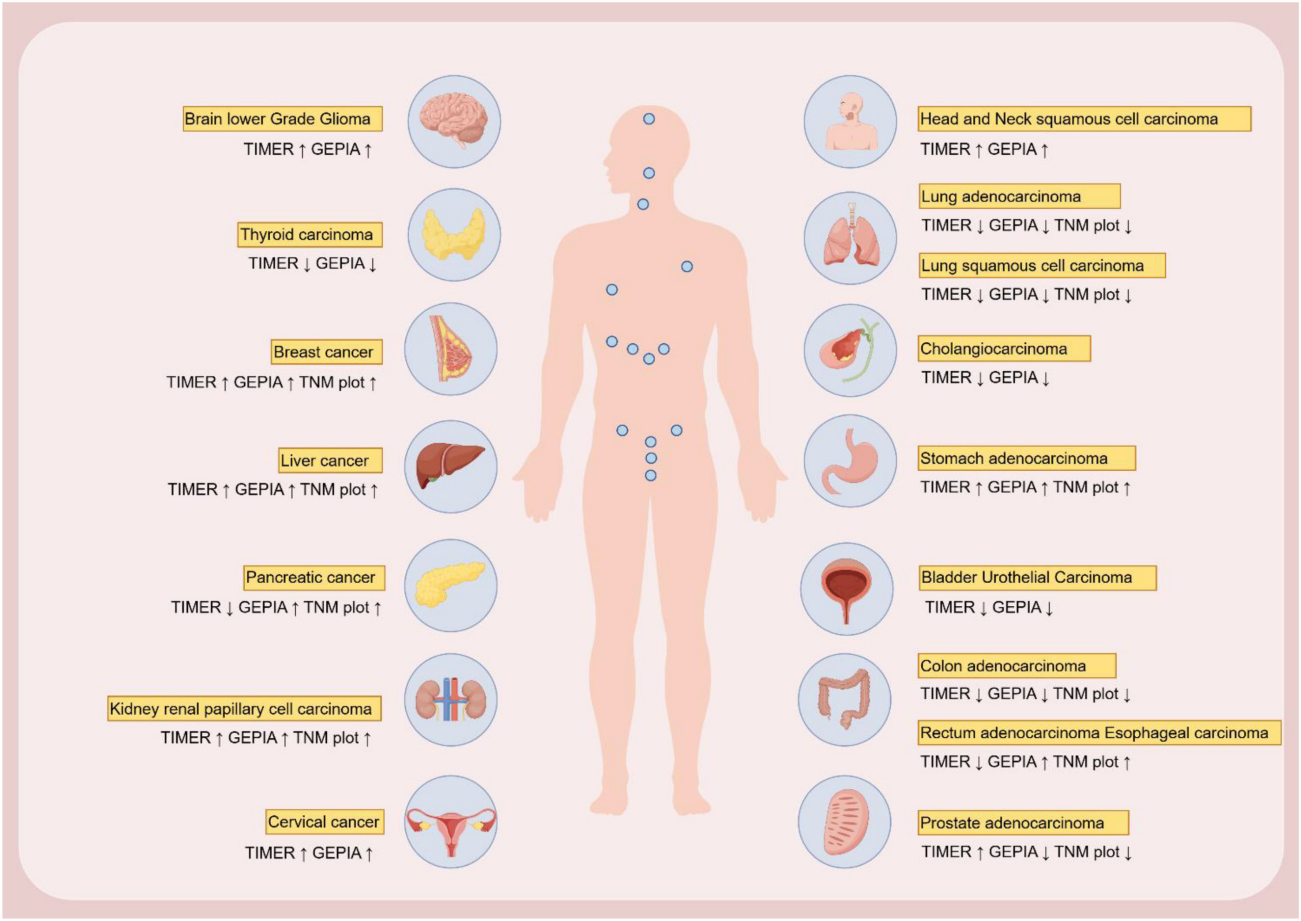
Integrin α L expression in different cancers in GEPIA, TNMplot and TIMER online databases.

The expression and function of Integrin α L may exhibit variability across different tumor types. This study provides a comprehensive summary of Integrin α L expression across various documented tumor types. Furthermore, the expression levels of Integrin α L in 31 distinct tumors were analyzed utilizing the GEPIA, TIMER, and TNMplot databases. Notably, discrepancies were observed in certain tumor types, where the expression of Integrin α L within the same cancer type differed across databases. For instance, in pancreatic cancer, GEPIA and TNMplot indicate elevated Integrin α L expression, whereas TIMER reports lower expression levels compared to normal tissue. Similarly, in prostate adenocarcinoma, GEPIA and TNMplot reveal high expression levels, while TIMER shows reduced expression relative to normal tissue. Potential explanations for these inconsistencies may include: 1) variations in cancer samples derived from different geographical regions or ethnic groups; 2) disparities in sample sizes between normal and cancerous subjects; 3) differences in detection methodologies and data processing techniques; and 4) tumor heterogeneity and tumor immune microenvironment heterogeneity. Both factors can lead to inconsistencies in gene expression profiles among cancer patients. Nonetheless, the data presented herein may offer valuable insights and a foundation for future investigations into the expression and functional role of Integrin α L in various cancers (**Figure 5**).

## 4 Relevant roles of Integrin α L in medical diseases

### 4.1 Integrin α L and atherosclerosis

In addition to extensive research on Integrin α L in the context of malignant tumors, there is an increasing body of evidence suggesting that Integrin α L plays a crucial role in the pathogenesis of atherosclerosis. Yang et al. (76) utilized machine learning algorithms to effectively identify Integrin α L as a signature gene for predicting the progression of atherosclerotic plaques among a diverse array of genes. Their findings revealed a significant correlation between the expression levels of Integrin α L and the extent of immune cell infiltration, as well as the expression of immune checkpoint molecules. This suggests that Integrin α L may influence both plaque progression and stability by modulating the immune response, thereby providing a novel perspective on the immunopathological mechanisms underlying atherosclerosis.

To further elucidate the role of Integrin α L in atherosclerosis, Zheng et al. (77) conducted a complementary study. The researchers identified differentially expressed genes (DEGs) by analyzing gene expression data from carotid atherosclerotic plaques and normal carotid tissues. They employed the single sample Gene Set Enrichment Analysis (ssGSEA) method to evaluate the level of immune cell infiltration. Through the construction of a weighted gene co-expression network analysis, they identified gene modules that were closely associated with key infiltrating immune cells and subsequently screened for hub genes within these modules using a Protein-Protein Interaction (PPI) network. Notably, Integrin α L emerged as a prominent candidate, indicating its potential involvement in the immune response and the process of plaque formation through intricate interactions with other immune cells and genes. However, these studies have yet to explore the specific signaling pathways and biological functions associated with Integrin α L, thus presenting an avenue for future research.

Zhou and his team (Zhou et al., 2022) adopted a different methodology by analyzing gene expression data from patients with aortic valve calcification and metabolic syndrome, constructing receiver operating characteristic (ROC) curves to evaluate the diagnostic potential of each gene. Their analysis not only confirmed Integrin α L as a potential diagnostic marker for aortic valve calcification in the context of metabolic syndrome but also demonstrated a close relationship between its expression levels and the disease state. Furthermore, the team conducted a comprehensive analysis of the signaling pathways associated with Integrin α L, which included cytokine-receptor interactions, Toll-like receptor signaling, chemokine signaling, TNF signaling, NK cell-mediated cytotoxicity, NF-κB signaling, IL-17 signaling, and pathways related to cell adhesion molecules.

Along the periodontitis–atherosclerosis axis, accumulating evidence links localized oral inflammation to systemic vascular pathology. Periodontal pathogen stimulation upregulates expression of Integrin α L, a core component of LFA-1. This upregulation facilitates neutrophil and monocyte recruitment to local inflammatory sites, thereby sustaining chronic inflammation. Systemically, elevated Integrin α L enhances immune cell infiltration into arterial walls, increases oxidative stress, and augments pro-inflammatory cytokine secretion. Concurrently, it promotes macrophage uptake of oxidized Low-Density Lipoprotein (oxLDL) and foam cell formation, collectively accelerating atherosclerotic plaque progression (78).

In familial hypercholesterolemia-associated atherosclerosis, Integrin α L has been identified as a pivotal gene whose expression correlates with macrophage uptake of acetylated LDL (AcLDL) and foam cell formation. These findings suggest that beyond mediating immune adhesion, Integrin α L may contribute to early atherogenesis by regulating macrophage lipid internalization and inflammatory activation (79). Clinically, monocytes from patients with early coronary atherosclerosis exhibit elevated Integrin α L expression. This molecule synergizes with adhesion molecules such as ICAM-1 to enhance monocyte adhesion to vascular endothelium and subsequent transmigration into the intima, establishing the cellular foundation for plaque initiation (80).

In parallel, Integrin α L has been identified as a critical mediator of non-classical monocyte (NCM) adhesion to the endothelium and patrolling activity throughout the peripheral vasculature. By facilitating NCMs to overcome the anti-adhesive glycocalyx of large vessels and establish close interactions with endothelial membrane-associated ligands (e.g., CX3CL1 and DLL1), Integrin α L dependent adhesion promotes the uptake of endothelial-derived CSF1, which is essential for NCM survival and homeostasis. Although atherosclerotic plaque formation was not directly investigated, these findings, together with prior evidence implicating NCM patrolling in early atherogenesis, suggest that Integrin α L may influence early arterial wall homeostasis by sustaining a competent NCM population for endothelial surveillance and clearance (81). These findings suggest that Integrin α L may play a significant role in the pathogenesis of valvular calcification and may also influence the progression and stability of atherosclerotic plaques by modulating immune responses and immune cell infiltration.

### 4.2 Integrin α L and systemic sclerosis

Systemic Sclerosis (SSc) is a multifaceted autoimmune disorder, and its pathogenesis is not yet fully elucidated. In their study, Wang et al. (82) investigated the expression of Integrin α L in CD4+ T cells from SSc patients using reverse transcription polymerase chain reaction (RT-PCR) and flow cytometry, while also exploring its potential mechanisms of action. The findings revealed a significant increase in Integrin α L expression levels in the CD4+ T cells of SSc patients compared to healthy controls. Additionally, a positive correlation was identified between the expression levels of Integrin α L and the severity of clinical disease activity. To further substantiate the role of Integrin α L in SSc, the researchers conducted co-culture experiments involving CD4+ T cells and antigen-presenting cells, B cells, or fibroblasts, both with and without anti- Integrin α L antibodies. They assessed the proliferation capacity of CD4+ T cells, the IgG production capability of B cells, and the expression levels of COL1A2 mRNA in fibroblasts. The results indicated that the inhibition of Integrin α L significantly reduced these biological processes, suggesting that Integrin α L is integral to the pathogenesis of SSc. However, additional in-depth studies are necessary to elucidate the mechanisms underlying the overexpression of Integrin α L in CD4+ T cells and its specific pathogenic role in SSc.

### 4.3 Integrin α L and gloomy

The potential antidepressant properties of statins, which are commonly prescribed for cardiovascular conditions, have garnered significant research attention. Jiang and his research team (83) undertook a comprehensive investigation to examine the off-target causal effects of statins on depressive risk and symptoms, focusing on the inhibition of Integrin α L and HDAC2, as well as traits linked to shared biological pathways identified through Connectivity Map (CMap) analyses. Utilizing advanced genetic and bioinformatics methodologies, the researchers analyzed an extensive dataset that included genotype, phenotype, and medication usage information from a substantial cohort of patients. The findings revealed a strong association between gene-mediated suppression of Integrin α L and the counts of basophils, monocytes, and neutrophils, suggesting a potential role for Integrin α L in the etiology of depression. Subsequent analyses further corroborated the causal involvement of Integrin α L in depression, indicating that it may serve as a viable therapeutic target. This discovery provides a significant theoretical framework and potential therapeutic approach for preclinical investigations and forthcoming clinical trials assessing the impact of statins on depressive disorders. Moreover, it underscores the importance of considering blood biomarkers in the exploration of the relationship between statins and depression.

### 4.4 Integrin α L and rheumatoid arthritis

Rheumatoid Arthritis (RA) is a chronic autoimmune disorder characterized by inflammation, pain, and dysfunction of the joints. Integrin α L, a member of the integrin family, has been identified as playing a significant role in the pathogenesis of RA. Research conducted by Fougerolles et al. (84) explored the function of Integrin α L in RA through antibody blockade experiments and knockout mouse models. Their findings indicated that Integrin α L is highly expressed in infiltrating inflammatory cells and joint tissues. Moreover, they demonstrated that the inhibition of Integrin α L function significantly mitigated the onset and progression of RA. The study also revealed that arthritis induced by antibodies against collagen resulted in a markedly lower severity of arthritis in α1 α L subunit knockout mice compared to wild-type mice. Additionally, the blockade of Integrin α L led to a significant reduction in inflammatory cell infiltration and lessened cartilage destruction in the joint tissues of arthritic mice, thereby underscoring the critical role of Integrin α L in the inflammatory response associated with RA. However, it is important to note that the primary focus of this investigation was on the role of Integrin α L in leukocyte recruitment and vascular endothelial adhesion, necessitating further research to clarify its potential mechanisms in RA.

In a subsequent study, Lowin and colleagues (85) conducted further investigations and observed that the expression levels of Integrin α L were significantly elevated in the synovial tissues of RA patients, showing a strong correlation with the degree of inflammation. The development of an Integrin α L knockout mouse model yielded significant results: these knockout mice exhibited a marked reduction in RA symptoms, including joint swelling, inflammatory cell infiltration, and cartilage destruction. Furthermore, synovial cells from Integrin α L knockout mice demonstrated a substantially reduced capacity to produce inflammatory factors. These findings not only reinforce the essential role of Integrin α L in the pathogenesis and progression of RA but also provide compelling experimental evidence for its potential as a therapeutic target.

**Figure 6:**
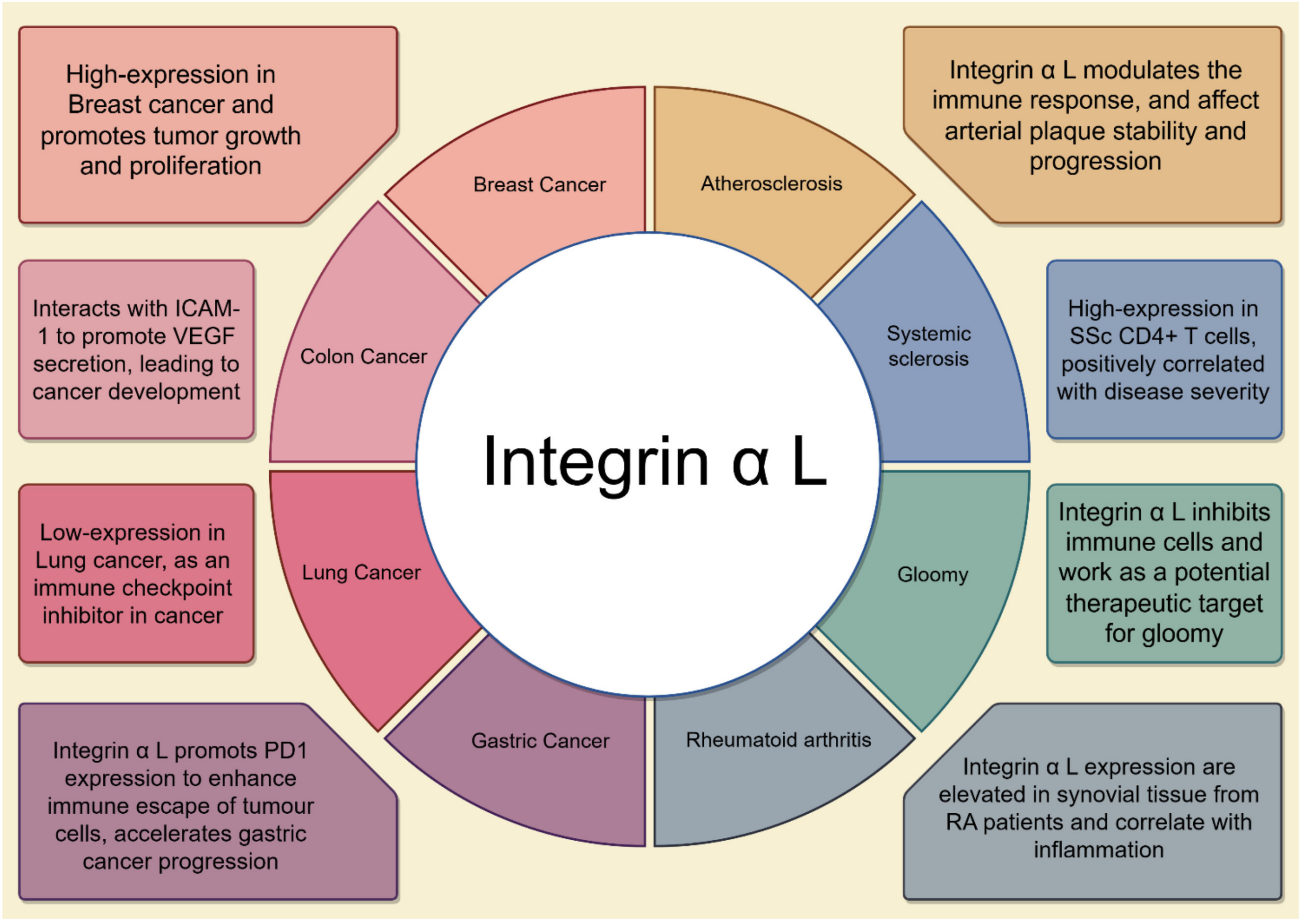
The emerging role of integrin α L in human cancer & non-cancer diseases.

Moreover, a growing body of omics-based and immune functional studies has revealed the multifaceted immunological roles of Integrin α L in RA. Bioinformatic multi-chip analyses have identified Integrin α L as one of nine hubs differentially expressed genes in RA, with its expression upregulated in the peripheral blood and synovial tissue of patients and positively correlated with follicular helper T cell (Tfh) infiltration. These findings suggest that Integrin α L may contribute to synovial inflammation and disease progression by promoting both the proliferation and differentiation of Tfh cells, thereby enhancing B cell activation and autoantibody production (78). Within the joint microenvironment, studies examining integrin expression on dendritic cells (DCs) show that inflammatory stimuli (e.g., LPS, IL-1, TNF-α) can modulate β2 integrin subunit (CD11a/CD11b) expression and activation state on DCs 3. Specifically, alterations in the surface expression levels of Integrin α L on DC subsets in RA are linked to altered DC functional states. While not explicitly described in the provided literature as shedding soluble forms, shifts in expression/activity could impact the stability of DC-T cell immunological synapses and potentially influence the balance between T cell activation and tolerance, suggesting that the role of Integrin α L on DCs in RA may involve immunoregulatory functions beyond purely pro-inflammatory activities (86). While the precise mechanisms by which Integrin α L operates in RA remain to be fully elucidated through further in-depth studies, these results undoubtedly open new avenues for the treatment of RA (**Figure 6**).

## 5 Integrin α L and small-molecule inhibitors

**Figure 7:**
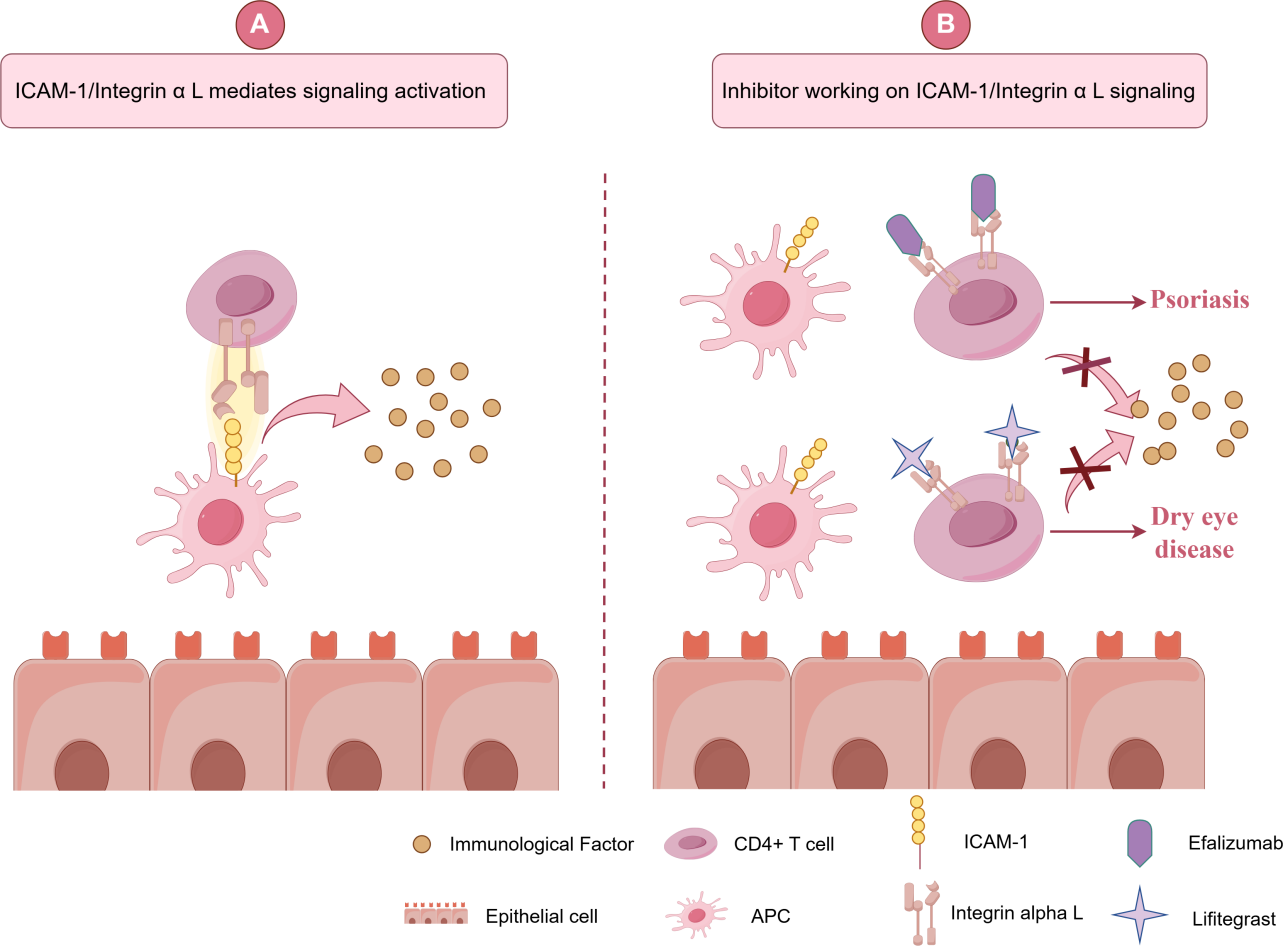
Application of Integrin α L inhibitors and related molecular mechanisms. **(A)** shows activation, where a CD4+ T cell interacts with an APC via ICAM-1 and Integrin α L, releasing immunological factors. **(B)** shows inhibition with drugs like Efalizumab and Lifitegrast, reducing signaling to treat psoriasis and dry eye disease.

In recent years, integrin inhibitors have garnered considerable attention as potential pharmacological targets. In 2003, efalizumab received marketing approval in the United States for the treatment of moderate-to-severe psoriasis. Efalizumab is a recombinant humanized monoclonal immunoglobulin antibody that targets CD11a, effectively blocking interactions with ICAM-1 and inhibiting T cell activation, transport, and adhesion to inflamed skin, thereby facilitating immunosuppression (87). However, its use was suspended due to adverse effects, including multifocal leukoencephalopathy. In 2016, Lifitegrast, which targets Integrin α L, was approved for the treatment of dry eye disease (88). Patients suffering from dry eye disease often exhibit increased expression of ICAM-1 and Integrin α L, with the interactions between Integrin α L and ICAM-1 promoting the migration of T cells to the inflamed conjunctiva and cornea. Numerous studies have indicated that the inhibition of integrin-ligand interactions may offer therapeutic benefits for this condition. Notably, in 2010, a research team led by Zhong (89, 90) conducted a study on integrin/ligand antagonists, identifying Lifitegrast as a significant drug candidate. Lifitegrast has been demonstrated to bind to structural domain I on LFA-1α, thereby competitively antagonizing ICAM-1 binding to Integrin α L. This mechanism inhibits T-cell recruitment to the cornea, resulting in a reduction of inflammation. Furthermore, the release of IFN-γ, IL, and other cytokines, which are positively correlated with the severity of dry eye, is concurrently inhibited. Consequently, integrin inhibitors have emerged as a crucial element in the management of immune and inflammatory diseases, providing substantial evidence to support Integrin α L as a promising therapeutic target for future interventions (**Figure 7**).

## 6 Research perspectives and challenges

In conclusion, the role of Integrin α L in various tumor types has been the focus of extensive research. However, the majority of existing studies concerning Integrin α L genes or proteins primarily rely on phenotypic observations or bioinformatics analyses. As a result, numerous questions remain unanswered regarding the specific kinases influenced by Integrin α L and their molecular mechanisms of action within the disease process, necessitating further investigation. To achieve a more comprehensive understanding of Integrin α L ‘s functions and mechanisms, it is essential to conduct in-depth studies that elucidate the precise mechanisms by which Integrin α L operates across different tumor types. This research should encompass, but not be limited to, an examination of Integrin α L ‘s specific effects on the tumor microenvironment and immune response, thereby clarifying its role in various disease contexts and revealing its diverse molecular mechanisms. Such insights will provide a more robust theoretical foundation for disease treatment.

Future research on Integrin α L may focus on several key areas: first, investigating the specific mechanisms by which Integrin α L contributes to tumor development and immune diseases, which could lead to the development of small molecule inhibitors and antibody drugs targeting Integrin α L to effectively impede tumor cell invasion, metastasis, and the release of immune factors. Additionally, research should explore Integrin α L’s role within the tumor microenvironment, aiming to modify its composition and function through the regulation of Integrin α L to inhibit tumor growth and metastasis. Furthermore, conducting clinical trials and validations for the therapeutic application of Integrin α L is crucial to assess its efficacy and safety. Overall, as a molecule of significant importance in various diseases, advancements in Integrin α L research offer valuable insights into the processes of disease development and metastasis, while also indicating new directions for future therapeutic strategies and targeted therapies. With the anticipated emergence and application of novel technologies such as precision medicine, multi-target combination therapies, innovative drug development, and gene editing, there is potential for the development of more effective therapies targeting Integrin α L, ultimately improving patient prognosis and quality of life.

In summary, this review has outlined the fundamental structure and function of Integrin α L, summarized the relationship between its expression patterns and functional properties with the invasive potential, metastatic capabilities, immune evasion strategies, and clinical outcomes of tumor cells and patients across various tumor types. Additionally, it has highlighted the significant role of Integrin α L in non-tumor diseases, including atherosclerosis, systemic sclerosis, depression, and rheumatoid arthritis. The review also assessed the feasibility of utilizing Integrin α L as a molecular marker for diagnosing certain diseases and tumors, which may yield new therapeutic targets for related conditions. Finally, it is anticipated that specific small molecule inhibitors will be identified or synthesized for the treatment of clinically relevant diseases.
